# Compassion fatigue among nurses in neonatal intensive care units

**DOI:** 10.1186/s12912-025-03385-2

**Published:** 2025-07-01

**Authors:** Linda Welde Hagen, I. Rød, N. M. Kynø, B. S. Tandberg

**Affiliations:** 1 Division of Paediatric and Adolescent Medicine, Department of Neonatal Intensive Care, Oslo University Hospital, Oslo, Norway; 2 Department of Master and Postgraduate Education, Lovisenberg Diaconal University College, Oslo, Norway; 3 Department of Health Sciences, Faculty of Medicine and Health Sciences, Norwegian University of Science and Technology, Ålesund, Norway; 4 Department of Nursing and Health Promotion, Acute and Critical Illness, Faculty of Health Sciences, Oslo Metropolitan University, Oslo, Norway; 5 Neonatal Intensive Care Unit, Department of Paediatric and Adolescent Medicine, Drammen Hospital, Vestre Viken Hospital Trust, Drammen, Norway

**Keywords:** Compassion fatigue, Qualitative research, Intensive care units, neonatal, Intensive care, neonatal, Neonatal intensive care nursing

## Abstract

**Background:**

Nurses in the neonatal intensive care units face continuous emotional demands as they care for critically ill infants and support distressed families. Repeated exposure to trauma and parental grief can lead to compassion fatigue – a state of emotional exhaustion that reduces the ability to empathize. The purpose of the study was to explore how nurses experience compassion fatigue while working in neonatal intensive care units.

**Methods:**

Qualitative, exploratory design. Eleven individual interviews with nurses and nurse specialists were conducted in August and September in 2023, at three neonatal intensive care units in three different hospitals in Norway. The reflexive thematic analysis described by Braun and Clark was used to analyze the findings. COREQ guidelines were used to ensure the quality of the reported data.

**Results:**

Two main themes with sub-themes reflecting the nurses` experiences were revealed. Theme 1: “The Voice of Compassion fatigue”, with sub-themes: “A double burden of care” and “The Silent Stray of Responsibility”, and Theme 2: “Perceived Consequences of Compassion Fatigue”, with sub-theme: “Reaching the Bottom of the Container of Care”. Perceived consequences affect the nurses physically and mentally and jeopardize their patient and private relationships.

**Conclusion:**

Neonatal Nurses experience compassion fatigue due to emotional strain and the hidden burden of caring for critically ill infants and their families. This may lead to physical and psychological exhaustion, affecting both patient care and personal life. These findings highlight the need for systematic support to safeguard nurses’ emotional well-being.

**Trial and protocol registration:**

Not applicable.

## Background

In professions such as nursing, a high degree of empathy and emotional engagement is required to provide care for patients and their families [1]. Compassion fatigue (CF) consists of the component’s burnout and secondary traumatic stress. Burnout may result in immediate reactions such as exhaustion, frustration, anger, and depression. Additionally, secondary traumatic stress can lead to experiences of negative emotions driven by fear and work-related trauma [2]. CF is described as the result of prolonged and overwhelming caring responsibilities, which can be triggered by repeated and unprocessed emotional engagement over time [3–5]. The work-related consequences include reduced decision-making and working capacity, a sense of helplessness, physical and emotional exhaustion, and a diminished ability to empathise and show compassion for patients and their families [4–7]. For individual nurses, CF can lead to impaired emotional regulation and a lack of self-care. This can, in turn, trigger physical complaints such as headaches, reduced sleep quality and lack of sleep, concentration problems and muscle pain. CF can be difficult for others to detect [4, 8, 9]. Unprocessed occupational stressors, including CF, may negatively affect patient safety and the physical and mental health of nurses, but also generate economic strain and lead to administrative challenges at the unit level [7, 9, 10].

Premature and critically ill infants are admitted to wards with high-intensity treatment and specialised nursing care. The care provided should be based on the fundamental principles of family-centered care (FCC). This means that the family is treated with dignity and respect, provided with comprehensible information, and empowered to maintain control and independence through participation in patient care and involvement in treatment [11–14]. Extensive medical and high-tech treatment of the infant may increase the risk of complications, physical damage and long-term illness. In some cases, the infant does not survive. High demands are placed on the quality of care, and on the nurse’s knowledge, in order to enable optimal development and health prospects for the infant [15]. The length of treatment in neonatal intensive care units (NICUs) can vary from a few hours to several days or even months. Nurses often follow a patient’s treatment over time. Continuity of care builds a firm foundation for a relationship between the nurse, the infant and the family.

NICU nurses have professional responsibility for the well-being of both the infant and the family during hospitalisation. FCC involves nurses providing parents with adequate psychological support to help them reduce stress and anxiety related to caring for their vulnerable baby [14]. Repeated exposure to parents’ emotional reactions, as well as the traumatic aspect of providing care to seriously ill and possibly dying infants, are viewed as demanding for nurses in NICUs. Overall, these factors may entail a risk of developing CF [5, 7, 10, 16, 17].

To our knowledge, there are currently few studies that examine how compassion fatigue affects nurses working in neonatal intensive care, both in their professional and personal lives. We therefore identify this as a gap in the existing research. The purpose of this study is to explore the experience of CF in NICU nurses with the following research questions:

- How do nurses in NICUs experience CF?
- What consequences does CF have for nurses working in NICUs?

## Method

### Design

The study has a qualitative, exploratory design. Data were collected using individual semi-structured in-depth interviews. Conceptualisation was achieved through reflexive thematic analysis according to Braun and Clark [18], and by developing codes and generating themes. Through the coding process, the themes were further defined. The Consolidated Criteria for Reporting Qualitative Research (COREQ) were used as a guideline to ensure the quality of the reported data [19].

### Participants

The participants in the study were nurses with a bachelor’s degree, nurses with post-graduate education and nurses with a master’s degree (in intensive, neonatal or pediatric care). Information about the study and an invitation to participate was sent via email to all nurses at three Norwegian NICUs to reach as many eligible informants as possible and to engage participants who could contribute reflections on their experiences. The information included consent form, formal request for participating including information on voluntariness, confidentiality and inclusion criteria, as well as the project plan. Inclusion criteria were more than two years of clinical practice in NICUs, and experience with both acute situations and longer patient pathways, preferably as a liaison nurse in a patient team. Participants who met the inclusion criteria and wanted to participate in the study expressed their interest directly to the first author or through the head nurse at the unit.

An interview guide was developed based on theoretical knowledge, research and the study aim (Table 1). The interview guide was pilot tested with one colleague to allow the first author to become familiar with the interview process and the equipment. The colleague was a nurse with a master’s degree and five years of experience working in a neonatal intensive care unit at the time. After the pilot, one question was revised because its wording was considered unclear. The pilot interview was not included in the data presented. Follow-up questions were based on the participants’ responses and narratives. All interviews were conducted with a digital audio recorder.

Data saturation was not predetermined in this study. In line with Braun and Clarke´s [18] guidance, small-scale projects such as this study typically benefit from conducting between six and ten interviews. Accordingly, the number of participants and interviews was deemed sufficient to identify patterns within the dataset, while remaining manageable enough to allow for a focused exploration of the nurses’ experiences and the study’s research questions. All nurses who contacted the research team to express interest in participating were included in the study.

**Table 1:** Excerpt from interview guide

Question	Example of follow-up questions
To what extent do you feel that CF applies to you?	1. You mentioned physical and mental health, how do you find that CF affects you physically and mentally? 2. How does it affect you at work? 3. How do you feel that this affects your private life?
Is CF something you talk about at work, and if so, in what way?	1. Who do you talk to when you feel worn out, fed up with work and exhausted of providing care? 2. Have you noticed any of the things we have been talking about now among your colleagues? 3. How do you notice that they might be tired of providing care?

### Analysis

The steps of Braun and Clarke’s [18] reflexive thematic analysis were followed. In the initial phase, we engaged deeply with the data to develop familiarity, and the material was transcribed after each interview and systematically reviewed. During the second phase, we generated codes and identified noteworthy features, which informed the development of preliminary themes in the third phase. The coding process was collaborative, involving discussions among the researchers to identify patterns relevant to the research question. Phases four and five, involving the review and refinement of themes, were conducted jointly. In the sixth phase, the first author prepared an initial draft of the manuscript, which was subsequently reviewed and developed collectively. Throughout the analysis, we repeatedly revisited the interview transcripts, maintaining a reflective stance toward both the participants’ voices and the text itself. This interactive process allowed for a deepening understanding of the data corpus with each successive reading [18]. The analysis programme NVivo was used in the systematic work with the codes [20]. The development of codes was a continuous process that was discussed in the research group. The codes were linked to descriptive statements in several rounds to capture the essence of the statements and codes [18]. A visual mind map was created with names of possible themes, after which all codes were collected under main themes and then divided into sub-themes that highlighted the essence of the data and created a relationship between the themes and existing knowledge [18]. This process was critically reviewed during the analysis by the research group. Extracts of the analysis process are shown in Table 2.

**Table 2:** Example of analysis process from codes to theme

Quote	Code Round 1	Code Round 2	Code Round 3 - Sub-theme	Theme
“We have patients who require a lot because they are very ill, combined with parents who are terribly scared. Then in fact you must deal with two different patient groups”	Intensive Care Patients requiring much for nurses Parents in crisis Manage care for two different groups of patients	Clinically complex patient group Working with parents places an additional strain on the nurses Parents reactions adds to the emotional burden	A Double Burden of Care	The Voice of Compassion Fatigue
“If the parents break down completely and start crying, you can’t say: I can’t handle this today, let me find a colleague. You have to stay there, take care of them and help them in every way you can. You just have switch on the button regardless of whether you have the energy for it”	Must be able to manage emotionally demanding situations Summon the strength to function at work Burden of responsibility	Capacity to endure and navigate difficult clinical encounters Capacity to engage professionally Overwhelming sense of responsibility	The Silent Strain of Responsibility	The Voice of Compassion Fatigue
“A day at work should allow you to still function when you get home. But you’ve often been through so much and given a lot of yourself at work, so when you arrive home, you’re told you’re grumpy. But I’m not grumpy; I just don’t feel like talking because I’m worn out”	Emotionally demanding Interferes with personal life Has no capacity left Feeling drained Engage in intensive caregiving during workhours Feeling empty when coming home	Emotional demands and workload significantly affect private lives Feeling mentally and physically exhausted Coming home emotionally drained	Reaching the Bottom of the “Container of Care”	Perceived Consequences of Compassion Fatigue

### Preconceptions and reflexivity

All the members of the research team have experience as nurses in NICUs. Despite cultural differences between NICUs, the authors’ prior understanding of medical language and terminology may have been an advantage during the interview situation and the analysis process. At the same time, there is a risk of preconceptions causing difficulties, as participants may experience situations and challenges differently from the authors interviewing them. This may prevent necessary follow-up questions from being asked. Preconceptions in the form of knowledge and personal experience may affect the research process, especially when the topic is sensitive. Therefore, critical reflection and repeated discussions between the authors was sought throughout the process. During the interviews, there was an emphasis on giving the informants enough time and space to reflect without being influenced by the interviewers. Rigour was ensured by employing Braun and Clarke’s six-phase approach to thematic analysis, with all interpretations of the data continuously subjected to internal discussion within the research team.

## Results

Eleven in-depth interviews were conducted in August and September 2023. The participants were five nurses with a bachelor’s degree and six with postgraduate education or a master’s degree. The interviews lasted 40–60 min and were conducted in designated meeting rooms within the department´s premises at the participants’ workplace. One interview was conducted via video call as it was not possible to arrange an in-person interview due to distance and availability of the participant. All participants were female, aged between 26 and 57 years, with an average of 13.5 years of experience in NICUs. They worked in level III and IV NICUs, representing various treatment categories related to the severity of illness and treatment needs, as well as the lower limit of prematurity based on gestational age. No participants withdrew from the study.

**Fig. 1:**
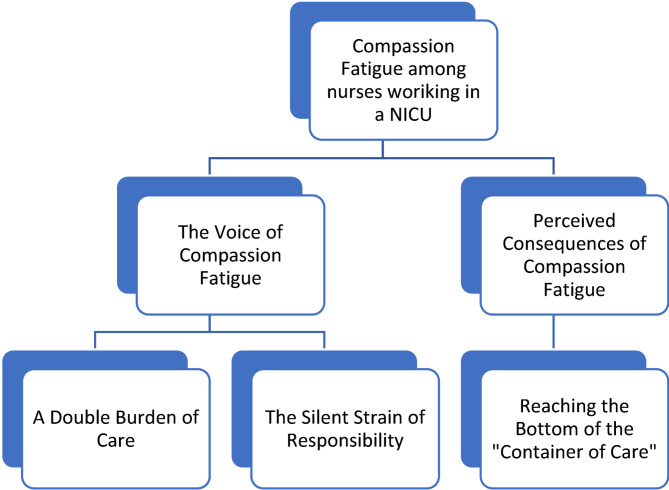
Overview of themes and sub-themes

The thematic map below shows how the three sub-themes formed the basis for the two main themes (Fig. 1). The results are presented under thematic headings with illustrative quotes.

### The voice of compassion fatigue

The first theme captures the nurses´ experiences of compassion fatigue. Their narratives reveal how they perceive the responsibility of caring for both the critically ill newborn and the infant’s parents. How the responsibility affected them was scarcely articulated in their working environment. The mental strain was felt to be hidden.

#### A double burden of care

The participants described experiencing a double burden of care in attending to both the sick infant and the parents. One participant elaborated: “We have patients who require a lot because they are very ill, combined with parents who are terribly scared. Then in fact you have to care for two different patient groups” (Participant 3). Moreover, the participants highlighted the challenge of navigating situations where their expertise and presence were in great demand, especially in palliative care and long patient pathways. When an infant is critically ill, the participants experience that parents have a vital need for emotional support and care at a vulnerable time. One participant explained: “There’s a big difference between patients. It’s a real strain to deal with complex patients and parents who demand a lot from you because they’re struggling. They kind of drain all your emotions because they need that support” (Participant 7).

The participants described how it was imperative at the beginning of the patient pathway to establish a relationship and foster trust to address the parents’ individual needs. This was felt to be a strain: “Building new bonds of trust can be rewarding. But sometimes it can drain you to get into those settings, get to know the relatives, and work to make them trust you” (Participant 10). Some participants regularly felt the need to distance themselves from situations that were mentally demanding, but they found it difficult to achieve this during a hectic working day. This was particularly evident in long care pathways. One participant expressed it as follows: “Sometimes it’s important to stop and say you need a break. Not because you don’t like the family, but because you need a little space” (Participant 11).

#### The silent strain of responsibility

Several participants reported feeling lonely in processing the emotional situations they experienced at work. They found it difficult to talk to their leader or colleagues for fear of being perceived as unsuitable for the job. Some individuals encountered difficulties in discussing matters with friends or family due to confidentiality obligations. Additionally, they considered that people who were not healthcare professionals might struggle to comprehend their experiences. One participant explained: “When I come home, and my husband asks me: How was your day? I say it was fine, and then I think about the baby who died. I can’t talk about it because he don’t understand what I go through” (Participant 6). The burden of handling the work-related emotional strains was considered silent and was perceived as lonely and hidden. In their daily work, the participants felt there was insufficient time and support to process the emotions aroused or their tough experiences. One participant elaborated: “We’re supposed to be professional, leave it behind when we go home, but it doesn’t work that way. We bring many situations home with us in our thoughts that we must process. We must process a lot on our own… unfortunately” (Participant 11).

The participants felt that their capacity for providing care at work was low during periods when they were trying to process previous stressful work-related events. They felt vulnerable to emotions and reactions from others. They found themselves caught between the expectations of employers and colleagues to perform effectively at work and their own limited capacity for self-care. One participant explained: “If the parents break down completely and start crying, you can’t say: I can’t handle this today, let me find a colleague. You must stay there, take care of them and help them in every way you can. You just have to switch on the button regardless of whether you have the energy for it” (Participant 7).

Some participants perceived that their extensive experience in the field gave them great responsibility for the infant and the parents. They often felt that their competence enhanced the quality of patient care, but also that the responsibility could be an additional and silent strain. One participant explained: “You can care for a very ill infant, perhaps with an inexperienced colleague. Then all the weight falls on you, and you feel a huge responsibility” (Participant 8).

### Perceived consequences of compassion fatigue

The second main theme addresses the consequences that participants experienced as a result of CF. Work-related experiences and impressions affected other aspects of their lives. Providing extensive care at work could lead to emotional distance, reduced cognitive capacity and a sense of emotional emptiness.

#### Reaching the bottom of the “container of care”

The participants described CF as depletion of their capacity to care, resulting in an empty “container of care”. One participant elaborated: “I believe that both privately and professionally, everyone has a kind of “container of care”. Sometimes it’s used more than other times. Sometimes in your private life, or at work. This container is small for some people and bigger for others” (Participant 5).

Participants found themselves becoming more engaged with certain infants and families, which they described as over-involvement. In a lengthy patient pathway, they felt that their “container of care” became empty, and they eventually distanced themselves emotionally from the infant and the family. When exhausted by empathy and with reduced emotional capacity, they avoided entering relationships and instead tried to keep busy with other tasks. One participant explained: “It got to be a bit too much, and you’re so tired that you really can’t go into a situation, whether it’s due to a challenging relationship or because you’re having a bad day yourself. You can’t go into it because you know it will drain you even more” (Participant 9). Another participant stated: “There’s a distance I hadn’t reflected on before. You protect yourself because getting involved with all the infants just kind of wears you out. You get mentally exhausted” (Participant 4).

Some participants had experienced periods where they lacked the energy to provide care at work. When their capacity to care was low, it affected how they related to the infant’s parents. One participant explained: “I’ve noticed that I get tired because I can’t handle the sadness or the stress, or when someone’s upset. I do the job, but I don’t have the emotional capacity to deal with them being very upset” (Participant 7). Several participants had found that their work performance had declined, leading to situations where patient safety was compromised. One participant explained: “It’s easy to make mistakes if your head’s not in the right place, and you’re tired and exhausted. Making medication errors or overlooking things you might have noticed if you were alert and focused” (Participant 1). According to the participants, reduced ability to think clearly and constructively, as well as decreased concentration, could be the result of an empty “container of care”. One participant elaborated: “I couldn’t do my work properly, memory loss (…). I had to write everything down and I was terrified of forgetting things completely. I spent a lot of energy on situations I usually handle well. (…). And it really wore me out” (Participant 11).

Participants felt that CF led to physical, mental or emotional distress. One elaborated: “If you have CF, you’re essentially burned out, but you just keep going because you’ve got used to being cynical and cold. You get a lot of adrenaline and joy from doing the job while you’re doing it. But you may not be the best version of yourself” (Participant 3). Additionally, participants described how the emotional strain of the job affected their relationships with family and friends. One participant explained: “A day at work should allow you to still function when you get home. But you’ve often been through so much and given a lot of yourself at work, so when you arrive home, you’re told you’re grumpy. But I’m not grumpy; I just don’t feel like talking because I’m worn out” (Participant 2). Another participant stated: “My CF has perhaps… My children have probably got what they needed, but my husband hasn’t, and I’ve felt like I’ve got no energy to socialise with friends because my job takes so much out of me” (Participant 6).

For some participants, the emotional strain of the job led to long-term sick leave, and some had considered seeking other, less demanding jobs. Several had long reflected on how the job affected them and had thought about how they could handle such difficult situations in the future. One participant explained: “Sometimes you ask yourself, is it worth it? Is this my life? Am I just supposed to be at work and then have no energy to do anything else? It’s just about focusing on the next shift and recharging” (Participant 10). Participants felt that they had repeatedly exceeded their coping capacity. One explained: “I don’t think it’s just when you don’t feel up to showing them things, advising them or being there. I think it’s also when you’ve actually managed to do a good job, but at the expense of yourself. And your ability to detach from what they [the parents] are going through” (Participant 3).

The participants all demonstrated professional and personal commitment to their work. This meant that they mobilized all their strength and care for the sake of the infants and parents. The consequence was reduced capacity in their private lives.

## Discussion

This study explores the experiences of CF of general and specialist nurses working in a NICU. The results confirm that CF is present among nurses working in NICUs. The findings illustrate the nurses’ feelings and experiences in a challenging work environment with a high workload.

Providing neonatal care led to a double burden for the nurses. An important point was establishing a good relationship with the parents at an early stage of the care process to enable the nurse to become a trusted, familiar person. A strong relationship between the nurse, the patient and the parents has been identified as a key factor in achieving successful patient and nursing outcomes [5]. This may help reduce the negative consequences of psychological stress, such as CF, among nurses in NICUs [5, 21]. Investing in new relationships with parents feels rewarding when successful [21, 22]. However, the close contact and attention to parents’ individual needs for support over weeks and months, and managing the emotions that arise, can be burdensome [21]. The nurses in this study confirmed that they viewed supporting parents as occasionally challenging. It has been described how nurses prioritise parents’ need for care over their own need for self-care [23]. Further, constantly dealing with the emotional burden of interaction with parents can lead to CF [17, 24].

Many participants found processing difficult situations to be a lonely and demanding task. However, social support from colleagues has a beneficial effect on mental health [16]. Such openness requires the security (a “safe zone”) to share difficult experiences and feelings [4]. At the same time, nurses find that being open about their experiences of stress at work may be perceived as not behaving professionally and as not coping with the job demands [25]. This is consistent with statements in our study, where some nurses reported being afraid that colleagues would consider them unsuitable for the job if they told them about situations they found difficult.

The nurses literally described their capacity for caring as “a container of care” that may also be emptied when CF occurs. Previous studies have found that intense involvement is required to work in the emotionally challenging environment of providing care to critically ill infants while also supporting their parents in crises and grief [21, 23]. This may result in a feeling of drained capacity for care, leaving the “container of care” empty, as described by the nurses in this study.

Coping with emotional demands at work was viewed by the participants as an expectation. Culture, norms and expectations can affect the individual’s ability to cope with situations, and the way in which emotional demands are acknowledged and handled [25]. Limited time and resources in everyday work gives nurses little opportunity to process the emotions that arise in patient situations, before they have to move on to the next emotionally challenging situation [8]. Different expectations and demands in the work environment lead to a perception of the emotional load as a tipping point, where contact and interaction with others become stressful [25]. Nurses are expected to be able to withstand the mental strain that follows from their work. They are left to navigate the exposure to stress and management of emotional demands on their own. These challenges could then become individualised and left to the individual to solve [25].

Several participants reported experiencing a variety of symptoms associated with CF. The condition may manifest as cynicism and emotional detachment, leading to impaired emotional regulation. This can affect the ability to show compassion and empathy towards the pain and strain of others [4, 7, 22]. This aligns with our findings, which indicate that the participants experienced a sense of indifference towards the effort they invested in building relationships. The intellectual impact of CF may lead to various errors, often due to reduced attention and poor concentration [8]. The participants noted that their capacity to work was diminished, which could compromise patient safety.

The participants in this study described how certain patient situations and relationships with families engage them far beyond what is expected. Over-involvement in patient care is described among nurses who develop CF, and can be exacerbated by exposure to unresolved stress or neglect of emotional needs over time [9, 17]. This can result in reduced reflection, where the ability to maintain necessary distance is suppressed [22]. However, it has also been previously reported that nurses experiencing CF distance themselves from patients [7, 26]. The participants in the present study also experienced periods of emotional distance, which led to their avoiding involvement with certain patient groups or relationships with parents.

Sick leave and thoughts of leaving their job were frequent among our participants. Nurses in NICUs use their energy in social interaction in an intense and demanding way, which results in an increased need for recovery and rest [22]. High emotional demands have been shown to increase the risk of long-term sickness absence [25]. Burnout is associated with an intention to quit, but does not actually make nurses leave their job [27]. The combination of high stress over time from relational and emotional events in everyday work and limited time for recovery and processing results in sick leave [25]. Nevertheless, it can be seen that CF and job satisfaction exist side by side. Nurses are satisfied with their work while also experiencing burnout and secondary traumatisation [3, 10]. The heavy workload also affected the nurses in our study in their private lives. When there is no longer enough room for social activities, friends and family, there is reason to believe that the emotional strain at work is too high and the cost to the individual is too great [22].

### Strengths and limitations

The participants represented NICUs in three hospitals with different treatment categories, subcultures and institutional contexts. However, the participants who volunteered to participate were probably those who had experienced CF, processed their experiences and had the energy and desire to talk about them. It is also possible that those who agreed to participate saw an opportunity to be heard.

Even though the findings revealed that CF existed among nurses in, it must be emphasised that not all NICU nurses will experience this. A nurse’s life situation, mental health and structural factors at the workplace may affect her or his experience of CF. In addition, it must be highlighted that CF, burnout and secondary traumatisation have overlapping symptoms, and may therefore have been confused by the participants.

To enhance the credibility of a study, it is important to choose the most appropriate method and a suitable number of interviews to obtain rich data that provide relevant answers to the research question. In this context, and due to the sensitivity of the topic, individual semi-structured interviews were considered to be an appropriate approach. The interviews provided a rich amount of data, although further interviews may have provided a broader and more nuanced picture. A weakness of the study may be that the interviews were conducted by LWH, NMK and BST, with varied experience of conducting qualitative interviews. This suggests that valuable data may have been lost during some interviews. A strength of this study was the research group’s reflexivity, involving repeated discussions during the analysis and coding of data from the interviews, as well as in the interpretations of the findings.

### Recommendations for future research

Nurses in Norwegian NICUs has in this study reported that they experience CF, yet the overall prevalence remains unknown. The nature of the patient population, the level of care provided, and broader structural conditions—such as economic constraints and resource availability—may significantly shape nurses’ daily work environment. Additionally, factors such as the unit’s care culture, nurses’ level of clinical competence, and the degree of leadership support may influence the development of CF. There is a need to explore how these contextual and organizational elements interact with the emergence of CF among NICU nurses. Given that Norway has a publicly funded and economically stable healthcare system, further research is warranted across diverse cultural and healthcare settings to better understand the phenomenon.

## Conclusion

The results of this study shed light on how nurses in NICUs experience the burden of care and the emotional strain of working with sick and dying infants and their parents. Nurses who experience great emotional strain in their work are at risk of developing CF, which can negatively impact both their professional responsibilities and personal lives. The experiences shared by the participating nurses indicate that CF is insufficiently addressed in clinical practice, leaving many to manage its effects on their own. These findings highlight the need for greater emphasis on the prevention and management of CF within neonatal intensive care units.

### Implications for policy and practice

The findings underscore the need for healthcare institutions to acknowledge CF as existing phenomena in NICUs, which potentially influence nurses working capacity significantly. Policy frameworks should prioritize the development of preventive strategies that address both individual and systemic contributors to CF. This includes providing access to clinical supervision and emotional support.

Educational institutions should integrate CF awareness and coping strategies into nursing curricula to better prepare future nurses for the emotional demands of neonatal care. Furthermore, leadership training for nurse managers should emphasize the importance of recognizing early signs of CF.

Given the possible influence of contextual factors such as economic resources, care culture, and leadership engagement, tailored approaches are necessary. Future policies should be informed by cross-cultural research to ensure relevance across diverse healthcare systems. Ultimately, addressing CF proactively can enhance nurse well-being, improve patient care quality, and contribute to the sustainability of the neonatal nursing workforce.

## Data Availability

The data that support the findings of this study are not openly available due to reasons of sensitivity and are available from the corresponding author upon reasonable request.
